# Study on synergistic inhibition and mechanism of flotation separation of fluorite and calcite by tannin and sodium humate

**DOI:** 10.1038/s41598-023-50233-x

**Published:** 2024-01-03

**Authors:** Zhi-xiong Zhu, Guang-Hua Nie, Yun Tang, Ying Jiang, Biyang Tuo, Jiaxin Li

**Affiliations:** 1 College of Mining, Guizhou University, Guiyang, 220025 China; 2 National & Local Joint Laboratory of Engineering for Effective Utilization of 3 Regional Mineral Resources from Karst Areas, Guiyang, 220025 China; 3 Guizhou Key Laboratory of Comprehensive Utilization of Nonmetallic Mineral Resources, Guiyang, 220025 China; 4 College of Mining, Guizhou University, Guiyang, 550025 China

**Keywords:** Environmental sciences, Chemistry, Engineering, Materials science

## Abstract

At present, the separation technology of fluorite and calcite is still immature, and the research in this paper can promote the improvement of the separation technology of fluorite and calcite. The selective inhibition mechanism of tannin and humate sodium on calcite was studied by means of actual ore flotation test, single mineral flotation test, Zeta potential measurement and FT-IR spectroscopy. The results show that the mixture of tannin and sodium humate inhibitor has a good inhibitory effect on carbonate under weak alkaline condition. The reaction products of sodium humate, tannin and calcium ions in solution interact with organic compounds adsorbed on the surface of calcite, forming multilayer adsorption on the surface of calcite, making calcite more hydrophilic. Based on density functional theory, Materials Studio (MS) was used to calculate the relevant adsorption energy, and the result was as follows: (a) compared with fluorite, tannin and humate sodium molecules are more easily adsorbed on the surface of calcite. (b) Compared with calcite alone adsorption of tannin molecules or sodium humate molecules, the adsorption state will be more stable, and the effect of tannin and sodium humate synergistic inhibition of calcite is better than the effect of inhibition alone. Therefore, using tannin and sodium humate as a combination inhibitor can effectively separate fluorite and calcite, which will promote the development and utilization of fluorite ore in industry.

## Introduction

The world is rich in total fluorspar resources, but is facing shortage of high quality mineral resources, such as China^1^. Carbonized fluorite is one of the main types of fluorspar, which is abundant, and the main gangue of this ore is calcite. Flotation is the most efficient method to recover this type of fluorite. Fluorite and calcite contain Ca^2+^. During flotation, the physical and chemical properties of the two minerals are very similar due to the interaction of dissolved minerals, dissolved ions and mineral surfaces. Both fluorite and calcite contain Ca^2+^, and collectors and inhibitors are more likely to interact with Ca^2+^ on the mineral surface, so collectors and inhibitors have similar effects on the surface of the two minerals, making it difficult to separate calcite from fluorite^2,3^. And the degree of difficulty in separation is closely related to the ratio of fluorite content to calcite mineral content. The smaller the ratio, the more difficult the separation of minerals. Generally, such fluorite deposits with ratios less than 4 to 5 are classified as refractory fluorite deposits^4,5^.

The flotation separation of fluorite and calcite has been increasingly studied. However, its flotation separation technology is not mature, especially for this kind of difficult ore with two very similar physical and chemical properties of minerals^6,7^. There have been some reports^8–11^ on the use of single or mixed acidic water glass, water glass, baked gum, sodium hexametaphosphate, caustic soda, citric acid and sodium lignosulfonate as calcite depressants in the flotation of fluorite ores at appropriate pH. It was shown that 2-phosphonobutane -1, 2, 4-tricarboxylic acid can selectively inhibit calcite at pH 9.0 under the condition of sodium oleate as a trapping agent, which also provides a new option for calcite depressants^12^.The study^13^ showed that using oleic acid as a trapping agent, the flotation performance of calcite in weak acid media was slightly worse than that of fluorite. If the appropriate inhibitor is combined with the collector, the grade and recovery of fluorite can be improved. In fluorite ore, appropriate inhibitors can selectively interact with the surface of calcite but not with the surface of fluorite, making the surface of calcite more hydrophilic, collectors can interact with the surface of fluorite but not with the surface of calcite, making the surface of fluorite more hydrophobic, so appropriate inhibitors and collectors are used in combination. It can effectively separate fluorite and calcite and improve the grade and recovery rate of fluorite. The flotation separation of carbonated fluorite ores will be a long-standing problem in fluorite flotation. Therefore, it is very important to carry out flotation separation studies of refractory carbonate fluorite ores for the development and utilization of such resources.

In this study, fluorite flotation tests were performed by using a mixture of tannin and sodium humate as a depressant of calcite. On this basis, the selective inhibition mechanism of tannin and sodium humate on calcite was studied by means of Zeta potential measurement and FT-IR spectroscopy.

## Materials and methods

### Materials

The pure mineral samples of fluorite and calcite were obtained from Guangzhou Mingfa Mineral Specimen Manufacturing Co., LTD. The samples of − 0.075 mm particle size and − 0.075 mm + 0.038 mm particle size were prepared by hand sorting, crushing, grinding and screening for pure mineral flotation test. − 0.038 mm sample size for infrared spectroscopy and other tests.

The multi-element chemical analysis of pure mineral samples of fluorite and calcite was carried out. The results are shown in Table 1 below.

**Table 1:** Multi-element chemical analysis of pure fluorite and calcite minerals/%.

Mineral	CaF	CaCO3	S	Si	Fe	Sr	Al
Fluorite	99.05		0.11	0.17	0.05	0.08	0.13
Calcite		98.98	0.16	0.14	0.082	0.17	0.15

It can be seen from the elemental analysis of Table 1 that the purity of fluorite and calcite is above 98%, and the purity of fluorite and calcite selected in the test meets the requirements of pure mineral test.

The actual fluorite ore was taken from Qingrong County, Guizhou Province, China. It was crushed and dry ground to − 2.0 mm. The results of chemical multi-element analysis of the samples are shown in Table 2.

**Table 2:** Results of chemical multi-element analysis of samples/%.

Constituent	CaF_2_	CaCO_3_	SiO_2_	Fe_2_O_3_	Al_2_O_3_
Content	45.75	18.02	26.57	1.81	1.53

Constituent	BaO	MgO	P	S	Others
Content	0.12	1.20	0.004	1.12	3.876

The results in Table 2 show that the CaF_2_ content in the ore is very low. The main components of gangue are CaCO_3_ and SiO_2_, whose content is as high as 47.59%, and the content of CaF_2_ is close. The ratio of CaF_2_ to CaCO_3_ is 2.64, which shows that the ore is insoluble fluorite ore.

The study of the mineral composition and the characteristics of the mineral assemblage showed that the main mineral in the sample was fluorite. The gangue minerals are mainly quartz and calcite, followed by pyrite, gypsum, barite, clay minerals, etc.

Sodium humate and tannin were used as flotation inhibitors. Sodium humate was composed of C_9_H_8_Na_2_O_4_ and the grade was analytically pure. It was purchased from Shandong Greenwater Chemical Co., LTD. The tannin composition is C_76_H_52_O_46_, the grade is analytically pure, and it was purchased from Nanhai Jiangshun Chemical Products Factory in Foshan City.

### Flotation test

The actual flotation test of fluorite ore was carried out at room temperature. The laboratory-prepared oleic acid (at a concentration of 1 mol/L) is heated and dissolved in water before being added to the flotation operation. Acidified water glass is configured according to the volume ratio of water glass 4: concentrated sulfuric acid 1: water 40. Other agents are chemical pure products. The actual ore flotation test uses XFDIV1.0 single cell flotation machine. After the flotation is completed, the floating products and the bottom products of the flotation cell are dried, weighed, calculated the yield and carried out chemical analysis to obtain the grade and recovery of fluorite.

Flotation exploration and rough conditions tests were performed using a mixture of sodium phosphate, sodium fluosilicate, tannin, sodium humate, tannin and sodium humate. For ease of expression, the mixtures of tannin and sodium humate are referred to as Df01 and Df02, respectively. Based on these tests, the effect of different types of depressants on flocculentation was tested at the dosage of 100 g/ton of depressant, 1000 g/ton of sodium carbonate, 300 g/ton of water glass, 300 g/ton of oleic acid and a grinding fineness of − 0.074 mm 75%.

### Single mineral flotation test

Single mineral flotation tests were carried out at room temperature using an XFGII tank flotation machine with a speed of 2010 r/min. Take 4 g of sample into the flotation cell for each test, add 40 mL of deionized water and stir for 1 min. Adjust the pH with hydrochloric acid or NaOH solution for 2 min. The concentration of HCl or NaOH used for pH regulation is 1 mol/L. Add the depressant sodium oleate at 2-min intervals. Manually scrape off the foam for 5 min. The product is dried and weighed. Calculation of mineral recovery by weight of product.

### Zeta potential measurements

The Zetasizer 2000 Zeta potential analyzer was used to determine the potential. Grind the sample with an agate mortar to – 5 μm. Place 50 mg of sample at a time in a small beaker, add deionized water, adjust the pH with HCl or NaOH, stir for 2 min, add the depressant and stir for 10 min. The potential was measured and repeated three times and the average value was taken.

### FT-IR spectroscopy

The NEXU-670 infrared spectrometer is used for infrared spectral analysis. Before the IR spectroscopy test, tannin and sodium humate solutions were prepared with deionized water and placed in 250 mL beakers, respectively, and the tannic acid solution was adjusted to neutral with 2% NaOH. The sample was ground to – 5 μm, added to a certain amount of the prepared solution, stirred for 10 min, let sit for 20 min, and the supernatant was separated. Then add deionized water and stir, let it stand for a certain time, and separate out the supernatant. Repeat the washing three times. Filtered through a conical funnel and washed three times with deionized water. The filtered solid was placed in a surface dish and dried naturally.

### Molecular simulation

Molecular simulation mainly involves constructing models at the atomic level of minerals with the help of computers, and then simulating the structure and motion of mineral molecules to further obtain various physical or chemical properties of the system under study. Compared to traditional theoretical and experimental studies, molecular simulations have the advantages of low cost, high safety, and the ability to present properties at the microscopic level. MaterialsStudio (MS) is a materials simulation software developed by BIOVIA. It includes various theoretical methods such as quantum mechanics, molecular mechanics and dynamics and Monte Carlo, and integrates as many as 23 functional modules such as CASTEP, DMol3, Forcite, and Sorption to enable cross-scale studies from microscopic electronic structure resolution to macroscopic performance prediction^14^.

The adsorption energy simulations obtained from MS simulations help to understand the adsorption mechanism of tannin and sodium humate molecules in calcite and fluorite, and provide theoretical support for efficient flotation separation of fluorite.

#### Calculation of adsorption energy

**Figure 1:**
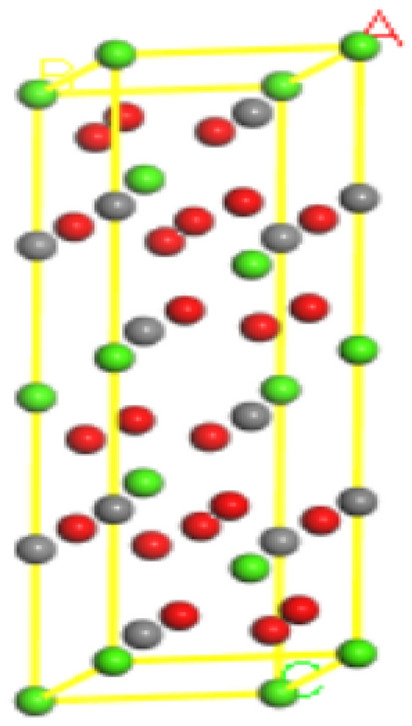
Optimized calcite model. Gray for carbon atoms, red for oxygen atoms, green for calcium atoms.

The crystal modeling of calcite was referred to the calcite crystal model in the Crystal Structure Database of the American Mineralogist, whose cell optimized prism lengths are a = 7.061 nm, b = 4.990 nm, and c = 4.990 nm, and the inter-prism angles are α = 90°, β = 60°, and γ = 120°, respectively, and the corresponding cell structures are shown in Fig. 1.

After calculations in the Morphology module of Materials Studio (MS) software, the most stable (1 − 1 2) surface of calcite was selected as the adsorption surface, and the tannin molecules were adsorbed after the steps of faceting, supercell and the establishment of vacuum layer, and the related energies were calculated.

**Figure 2:**
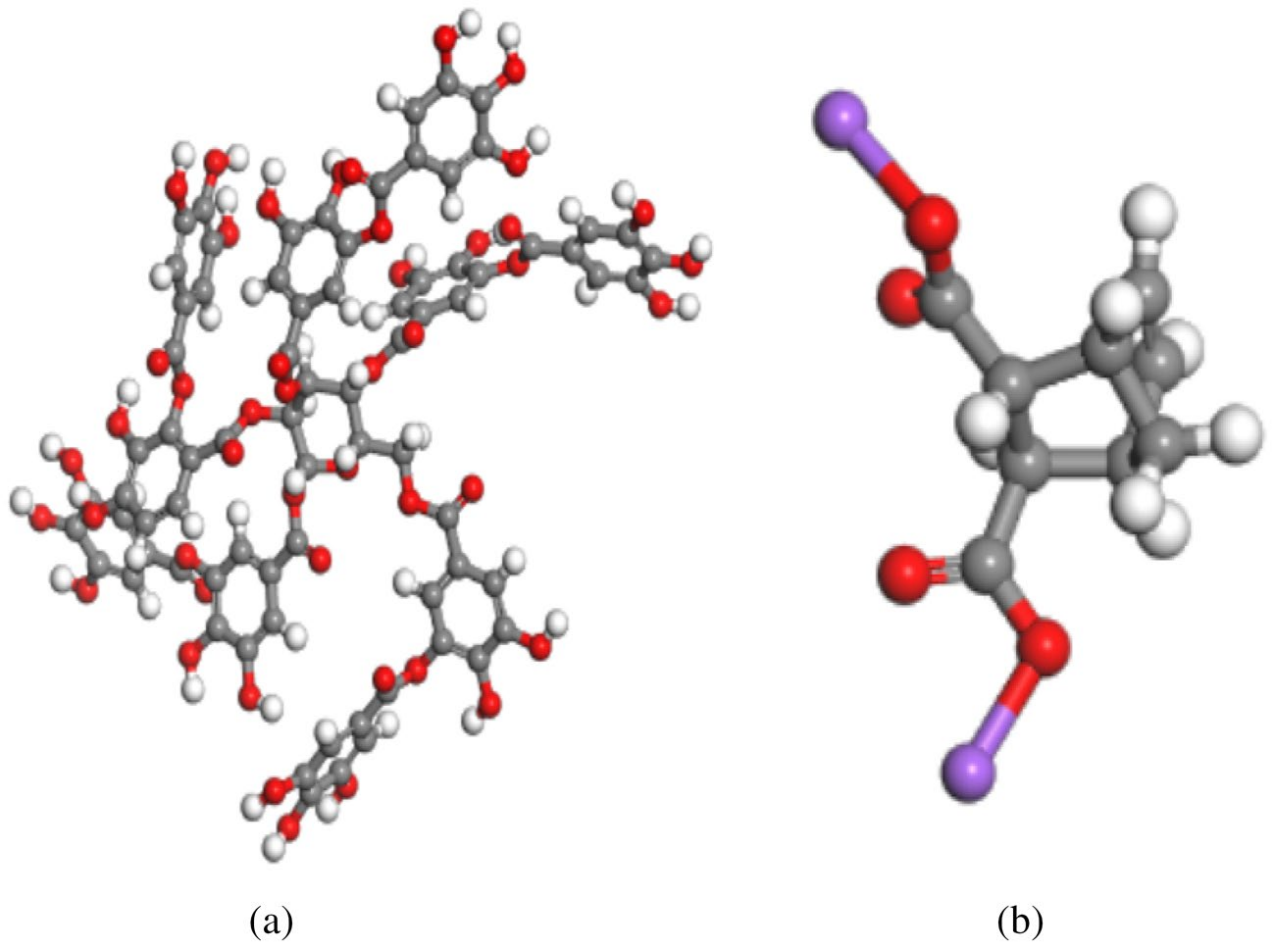
Optimized conformation of tannin and optimized conformation of sodium humate. (**a**) Optimized tannin model. Gray is carbon atom, white is hydrogen atom, red is oxygen atom. (**b**) Optimized model of sodium humate. Gray is carbon atom, white is hydrogen atom, red is oxygen atom and purple is sodium atom.

**Figure 3:**
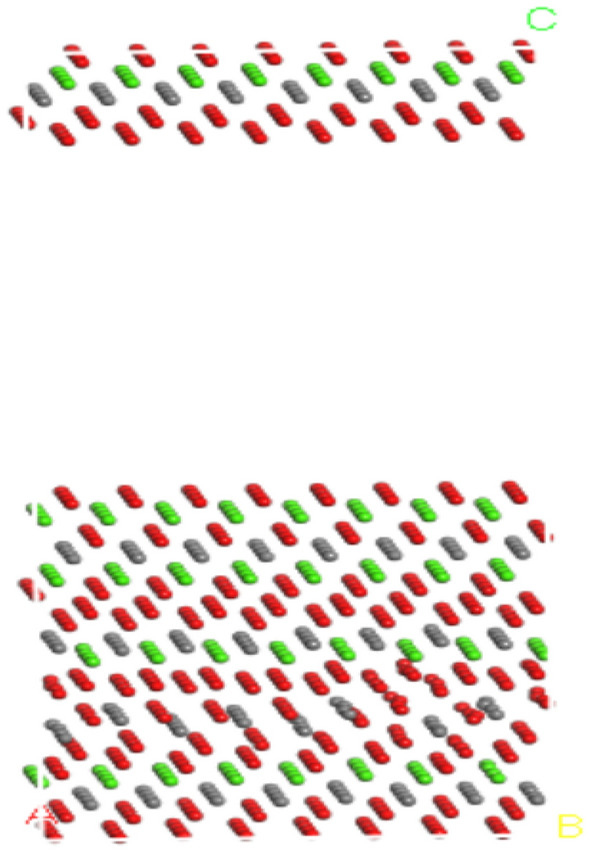
Optimized calcite (1 **− **1 2) surface model. Gray is carbon, red is oxygen, and green is calcium.

The optimized conformation of tannin and the optimized conformation of sodium humate are shown in Fig. 2, and the surface model of calcite (1 − 1 2) after structural optimization is shown in Fig. 3.

**Figure 4:**
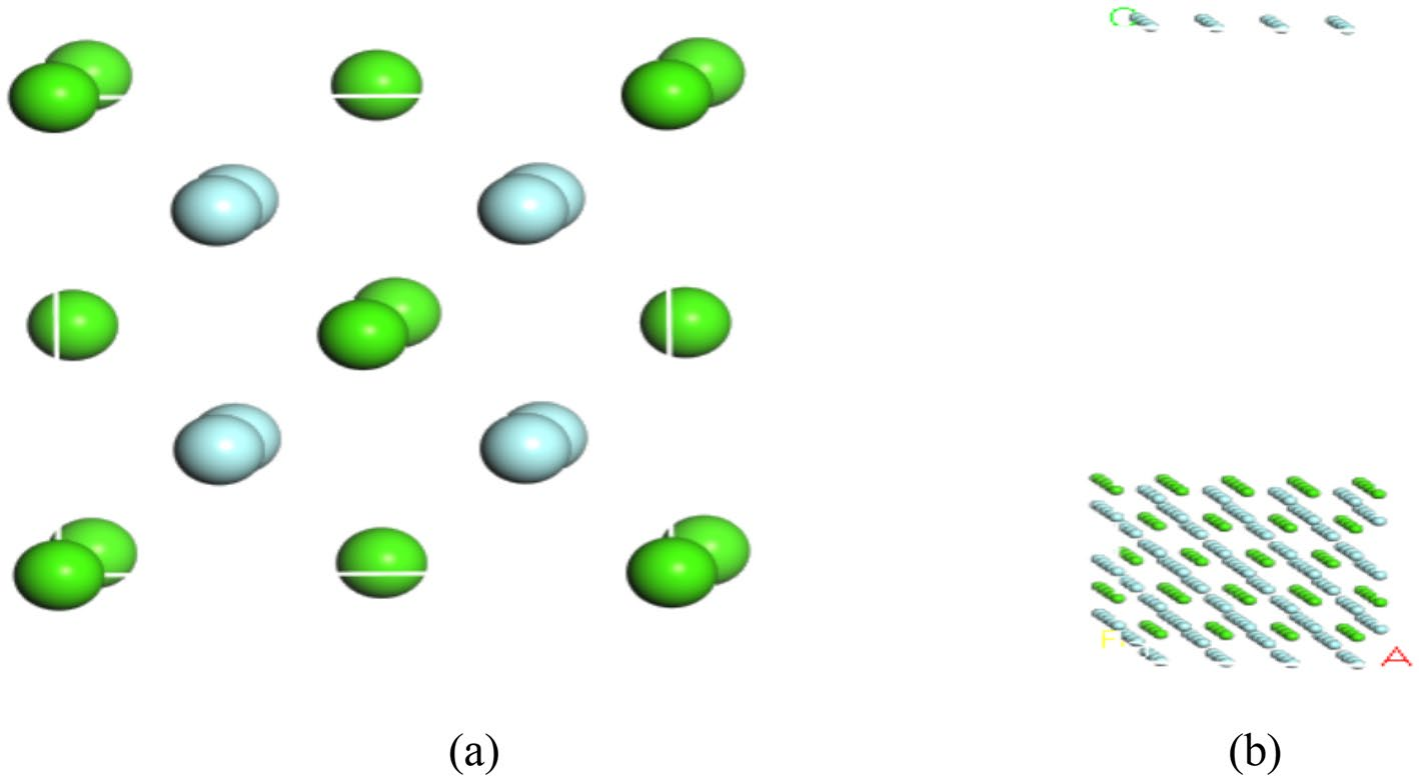
Crystal model of fluorite with optimized surface model of fluorite (1 1 1). (**a**) The optimized fluorite crystal model. The green ones are calcium atoms and the light blue ones are fluorine atoms. (**b**) Optimized fluorite (1 1 1) surface model. The green color is calcium atoms, and the light blue color is fluorine atoms.

The same calculation method and calculation steps were used to calculate the adsorption energy of fluorite for tannin adsorption with sodium humate. The crystal modeling of fluorite was referred to the fluorite crystal model in the American Mineralogist Crystal Structure Database, and its cell was optimized with prism lengths of a = b = c = 5.407 nm and inter-prism angles of α = β = γ = 90, respectively, and the corresponding cell structure is shown in Fig. 4. After the calculation of Morphology module of Materials Studio (MS) software, the most stable (1 1 1) surface of fluorite was selected as the adsorption surface. The crystal model of fluorite with the optimized surface model of fluorite (1 1 1) is shown in Fig. 4.

The positive or negative adsorption energy can visually represent the stability of the system after adsorption of foreign impurities. If the adsorption energy is less than zero, it means that the system releases energy when adsorbing foreign impurities and the system is stable after adsorption; if the adsorption energy is greater than zero, it means that the system absorbs energy when adsorbing foreign impurities and the system is unstable after adsorption^15^. When it is negative and the absolute value is larger, the easier the adsorption process is and the correspondingly more stable the system after adsorption^16^. The adsorption energy is calculated by subtracting the energy of each part before adsorption from the energy of the system after adsorption. The expression of adsorption energy is as follows:

1 $$ {\text{E}}_{{{\text{ads}}}} = {\text{ E}}_{{{\text{calcite }} + {\text{ tannin}}}} - {\text{E}}_{{{\text{calcite}}}} - {\text{E}}_{{{\text{tannin}}}} , $$

2 $$ {\text{E}}_{{{\text{ads}}}} = {\text{ E}}_{{{\text{calcite }} + {\text{ sodium humate}}}} - {\text{ E}}_{{{\text{calcite}}}} - {\text{ E}}_{{{\text{humate}}}} , $$

3 $$ {\text{E}}_{{{\text{ads}}}} = {\text{ E}}_{{{\text{fluorite }} + {\text{ tannin}}}} - {\text{E}}_{{{\text{fluorite}}}} - {\text{E}}_{{{\text{tannin}}}} , $$

4 $$ {\text{E}}_{{{\text{ads}}}} = {\text{ E}}_{{{\text{fluorspar }} + {\text{ sodium humate}}}} - {\text{ E}}_{{{\text{fluorspar}}}} - {\text{ E}}_{{\text{sodium humate}}} , $$

5 $$ {\text{E}}_{{{\text{ads}}}} = {\text{ E}}_{{{\text{calcite }} + {\text{ sodium humate }} + {\text{ tannin}}}} - {\text{ E}}_{{{\text{calcite}}}} - {\text{ E}}_{{{\text{tannin}}}} - {\text{ E}}_{{\text{sodium humate}}} , $$

In the formula: E_ads_ is the adsorption energy; E_calcite + tannin_ is the total energy of calcite (1 − 1 2) after surface adsorption of tannin; E_calcite + sodium humate_ is the total energy of calcite (1 − 1 2) after surface adsorption of sodium humate; E_fluorite + tannin_ is the total energy of fluorite (1 1 1) after surface adsorption of tannin; E_fluorite + sodium humate_ is the total energy of fluorite (1 1 1) after surface adsorption of sodium humate. E_calcite + sodium humate + tannin_ is the total energy of calcite (1 − 1 2) after surface adsorption of sodium humate and tannin; E_calcite_ is the energy of calcite crystals; E_fluorite_ is the energy of fluorite crystals; E_tannin_ is the energy of tannin molecules; E_sodium humate_ is the energy of sodium humate molecules.

Table 4 shows the adsorption energies of calcite (1 − 1 2) surface and fluorite (1 1 1) surface after adsorption of tannin and sodium humate molecules, respectively, and Table 5 shows the adsorption energies of calcite (1 − 1 2) surface after adsorption of tannin and sodium humate molecules, respectively, and the adsorption energies of calcite (1 − 1 2) surface after adsorption of both tannin and sodium humate.

**Figure 5:**
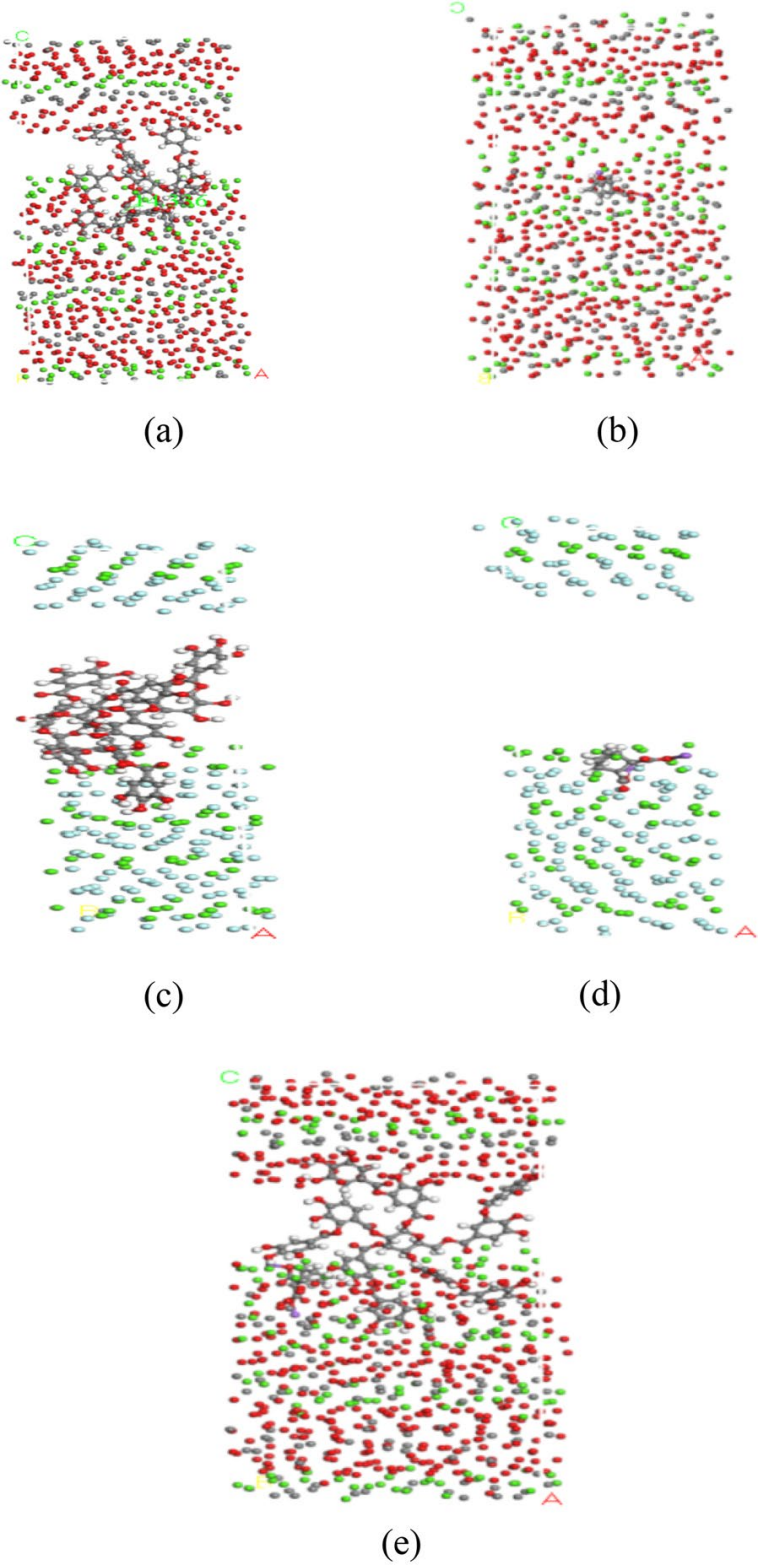
The optimized molecular adsorption model. (**a**) Molecular model for the optimized calcite (1 − 1 2) surface adsorbed tannin; (**b**) molecular model for the optimized calcite (1 − 1 2) surface adsorbed sodium humate; (**c**) molecular model for the optimized fluorite (1 − 1 2) surface adsorbed tannin; (**d**) molecular model for the optimized fluorite (1 − 1 2) surface adsorbed sodium humate; (**e**) molecular model for the optimized calcite (1 − 1 2) surface molecular model for adsorption of tannin and sodium humate.

The optimized molecular model of calcite (1 − 1 2) surface adsorbed tannin, the optimized molecular model of calcite (1 − 1 2) surface adsorbed sodium humate, the optimized molecular model of fluorite (1 − 1 2) surface adsorbed tannin, the optimized molecular model of fluorite (1 − 1 2) surface adsorbed sodium humate, and the optimized molecular model of calcite (1 − 1 2) surface adsorbed tannin and sodium humate are shown in Fig. 5.

## Results

### Flotation test

1. The effect of different types of depressants on flotation.

**Figure 6:**
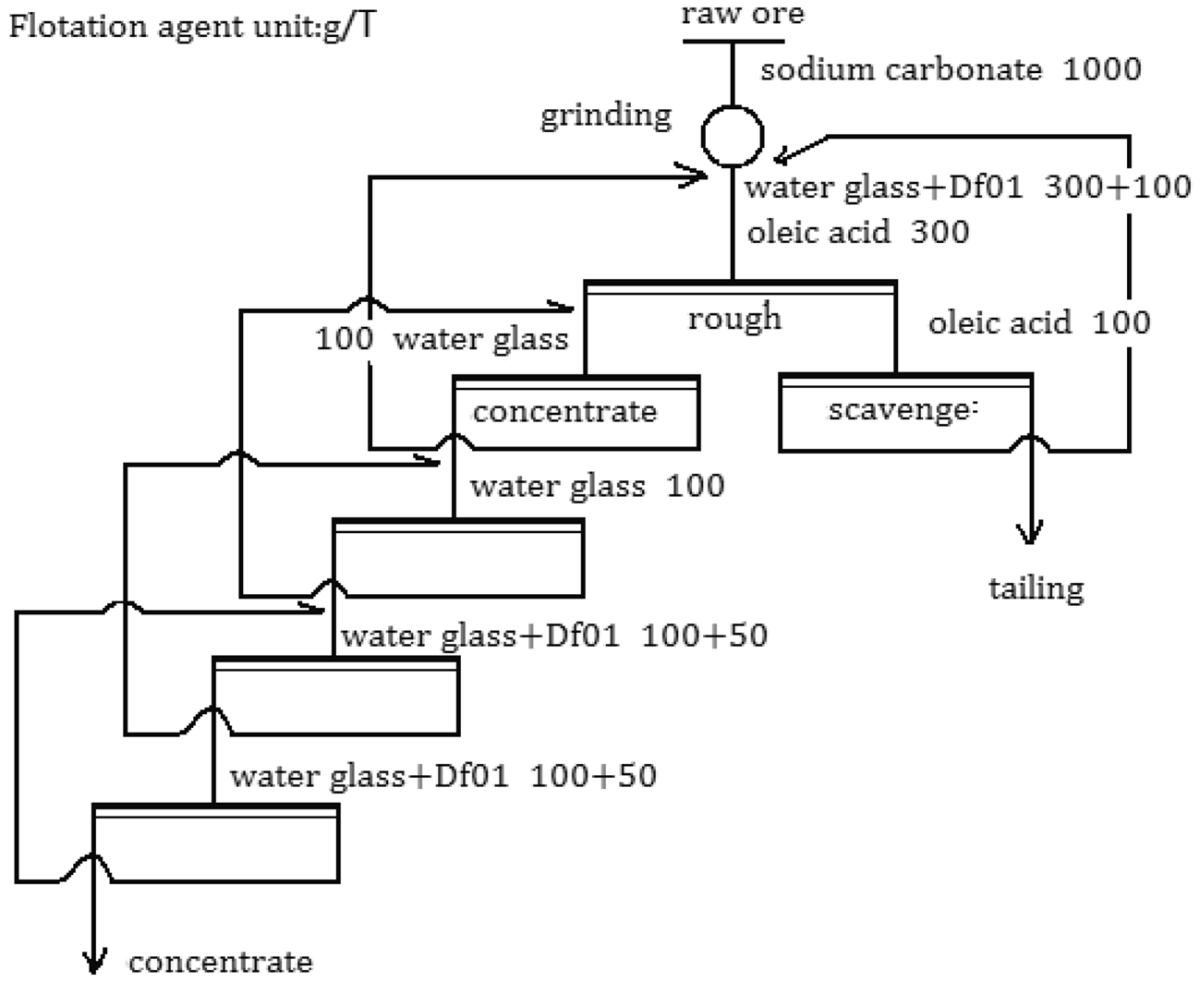
Flowsheet of close-circuit floatation.

The test flow and flotation agent scheme are shown in Fig. 6. Some of the test results are shown in Table 3.

**Table 3:** Test results of different kinds of depressants on flotation/%.

Depressant	Product	Yield	Grade	Recovery
Not added	Rough concentrate	57.69	74.06	93.10
	Tailings	42.31	7.48	6.90
	Primary sample	100.00	45.89	100.00
Tannin	Rough concentrate	48.12	75.32	80.02
	Tailings	51.88	17.44	22.07
	Primary sample	100.00	45.29	100.00
Sodium humate	Rough concentrate	56.23	75.28	93.19
	Tailings	43.77	7.07	6.12
	Primary sample	100.00	45.42	100.00
Sodium phosphate	Rough concentrate	52.21	73.10	83.29
	Tailings	47.79	16.02	16.71
	Primary sample	100.00	45.82	100.00
Sodium fluosilicate	Rough concentrate	55.08	74.68	90.38
	Tailings	44.92	9.75	9.62
	Primary sample	100.00	45.51	100.00
Df01 (Tannin + sodium humate = 1:5)	Rough concentrate	52.21	79.82	91.01
	Tailings	47.79	8.61	8.99
	Primary sample	100.00	45.79	100.00
Df02 (Tannin + sodium humate = 1:3)	Rough concentrate	52.65	78.56	90.61
	Tailings	47.35	9.05	9.39
	Primary sample	100.00	45.65	100.00

The results in Table 3 show that the recovery of CaF_2_ was 91.01% with a grade of 79.82% when a mixed depressant Df01 (1:5 mixture of tannin and sodium humate) was used for the crude float. The flotation result was the best. When sodium phosphate was used as a depressant, the flotation result was the worst, followed by sodium fluorosilicate. When tannin or sodium humate was added, the CaF_2_ grade of fluorite crude concentrate was improved. The recovery of CaF_2_ was lower when tannin was added and higher when sodium humate was added, which indicated that the depressant of tannin was stronger than other depressants and the selectivity of sodium humate was better than other depressants.

**Figure 7:**
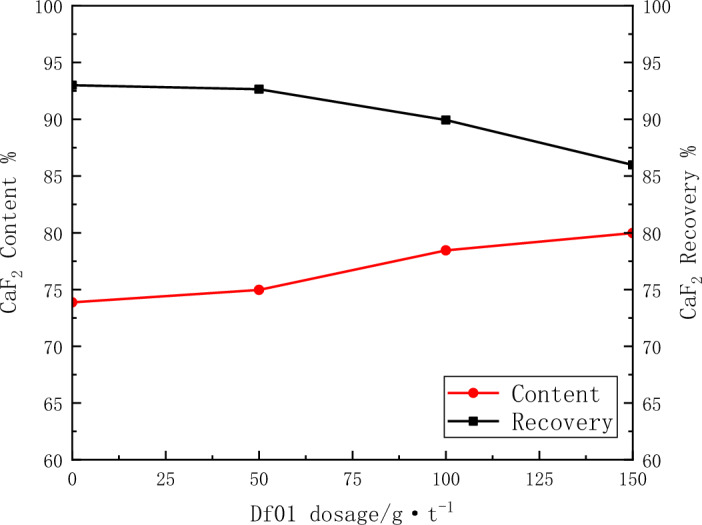
Effect of the amount of Df01 on flotation.

The depressant effect of the mixture of tannin and sodium humate was the best. the test results of Df01 dosage on the flotation effect are shown in Fig. 7.

As can be seen from Fig. 7, the concentrate grade increases significantly with the increase of Df01 dosage, but it also has a greater impact on the concentrate recovery. Especially when the dosage exceeds 100 g/t, the recovery rate is greatly reduced.

2. Closed circuit flotation test.

Based on rough condition tests and process tests, closed-circuit tests were conducted. The closed circuit flotation flow chart and flotation reagent scheme are shown in Fig. 6. The results are shown in Table 4. The results show that the final fluorite concentrate CaF_2_, CaCO_3_ and SiO_2_ content indexes meet the three grade standards for the chemical requirements of fluorite concentrate. It can be seen that good indicators can be obtained with this process.

**Table 4:** Results/percentages of closed-circuit floatation tests/%.

Products	Yield	Taste			Recovery rate		
		CaF_2_	CaCO_3_	SiO_2_	CaF_2_	CaCO_3_	SiO_2_
Concentrates	43.77	95.52	1.31	1.07	91.20	3.19	1.76
Tailings	56.23	7.18	30.92	46.46	8.80	96.81	98.24
Raw Ore	100.00	45.85	17.96	26.59	100.00	100.00	100.00

### Single mineral flotation test

**Figure 8:**
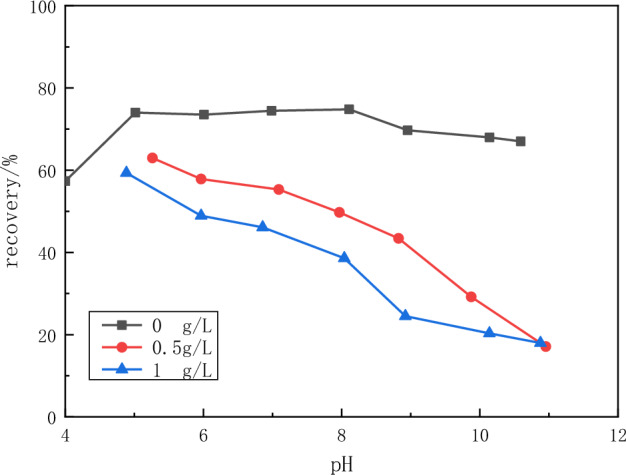
Flotation recovery of fluorite as a function of pH of tannin concentration.

The effect of tannin and sodium humate on the flotation of fluorite and calcite minerals was investigated. From Fig. 8, it can be seen that tannin has a depressant effect on the flotation of fluorite. This effect increases with increasing pH and with increasing tannin concentration. Due to tannins, the recovery of fluorspar decreased from nearly 80% to less than 60% when the pH was 5. When the pH is 11, the recovery of fluorspar drops to less than 20%.

**Figure 9:**
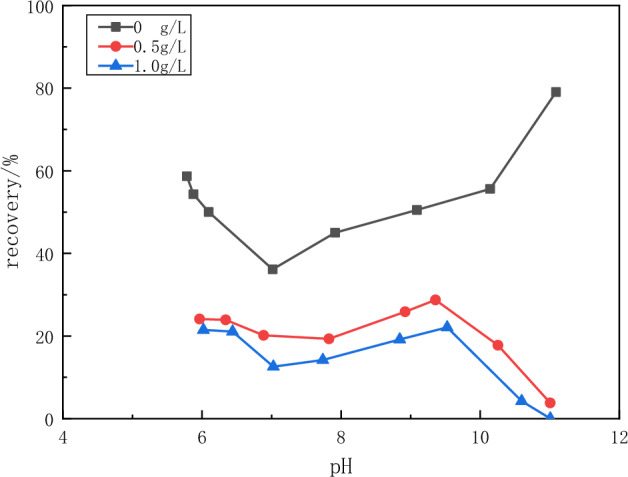
Flotation recovery of calcite as a function of pH of tannin concentration.

As can be seen from Fig. 9, the recovery of calcite was less than 20% at around pH 7 under the depressant of tannins. The recovery increased with increasing pH, reaching the highest value near pH 9. The recovery of calcite decreases linearly when the pH is greater than 9, approaching 0 at around pH 11.

**Figure 10:**
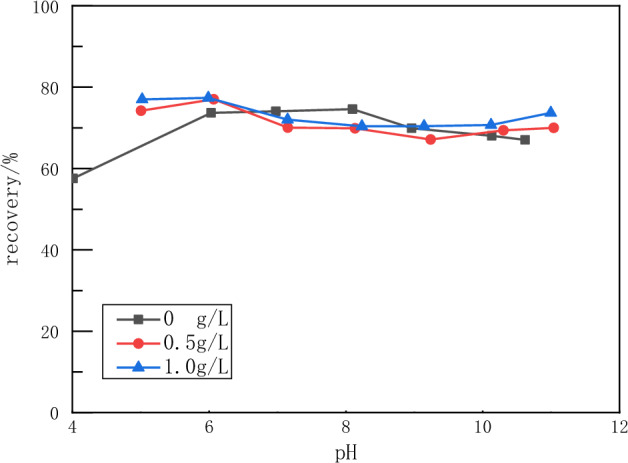
Flotation recovery of fluorite versus pH of sodium humate concentration.

**Figure 11:**
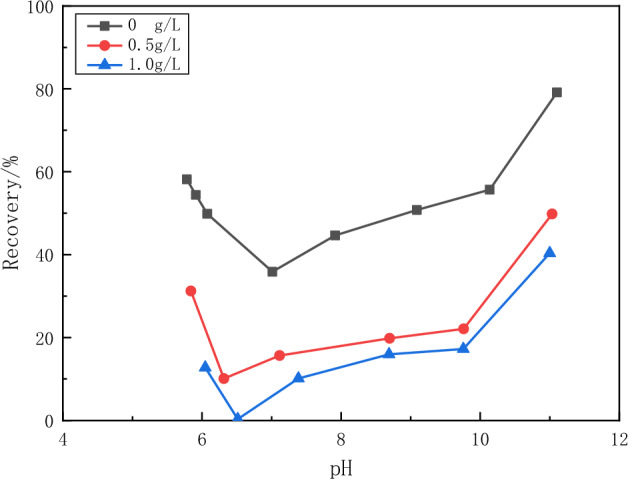
Flotation recovery of calcite as a function of pH of sodium humate concentration.

As can be seen in Fig. 10, sodium humate has little effect on the flotation of fluorite. The flotation recovery of fluorite did not vary much with the concentration of sodium humate. It can be seen from Fig. 11 that sodium humate has a strong depressant effect on the flotation of calcite. In the pH range of 6–10, the recovery of calcite decreased from more than 40% to less than 20% with the addition of sodium humate. The recovery decreased with the increase of sodium humate concentration. The recovery of calcite was 0 when the tannin concentration was 1 g/L and the pH value was around 6.5.

The above experimental results show that tannins can inhibit both minerals under alkaline conditions with pH values greater than 9. In the pH range of 6–8, tannins inhibited calcite more than fluorite. Sodium humate has a strong depressant effect on calcite and little depressant effect on fluorite flotation. Therefore, sodium humate has a good selective depressant effect on calcite.

**Figure 12:**
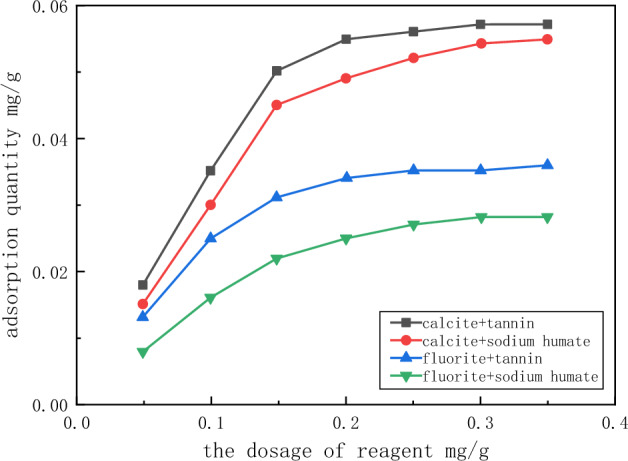
Relationship between reagent dosage and adsorption of fluorite and calcite.

The relationship between the amount of tannin, the amount of sodium humate and the adsorption of fluorite and calcite was investigated at pH 8, and the results are shown in Fig. 12. The results were that the adsorption capacity of calcite for tannin and sodium humate was greater than that of fluorite, and the adsorption amount increased with the increase of reagent dosage. However, when the amount of tannins exceeded 0.15 mg/g, the adsorption of tannins on minerals increased very little. At this time, the adsorption amount of calcite for tannin was about 0.055 mg/g, and that of fluorite was about 0.033 m/g. When the amount of sodium humate exceeded 0.15 mg/g, the adsorption increase rate of sodium humate was low. At this time, the adsorption amount of calcite to sodium humate was about 0.045 mg/g, and that of fluorite was about 0.022 mg/g.

### Zeta potential measurement

**Figure 13:**
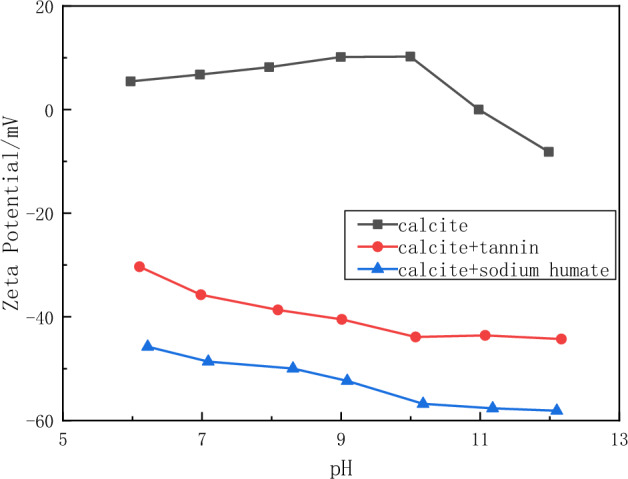
Zeta potential of calcite in the absence and presence of reagents as a function of pH.

The effect of tannin and sodium humate adsorption on the calcite surface charge was investigated by measuring the potential. The results in Fig. 13 show that the zeta potential on the calcite surface is negative with the addition of sodium humate and tannin, and the negative value increases with the increase of pH. The surface of calcite is negatively charged when the pH value is greater than 11. After the addition of sodium humate and tannins, the adsorption of sodium humate and tannins significantly reduced the surface potential of the minerals. This is because before the addition of tannin and sodium humate, the surface of calcite is covered with metal cations such as Ca^2+^, making the surface of calcite positively charged, after the addition of tannin and sodium humate, tannin and sodium humate interact with metal cations such as Ca^2+^ on the surface of calcite, reducing the number of cations on the surface of calcite, making the surface of calcite negatively charged. Therefore, the adsorption of sodium humate and tannins on calcite surface is chemisorption.

### FT-IR spectroscopy

**Figure 14:**
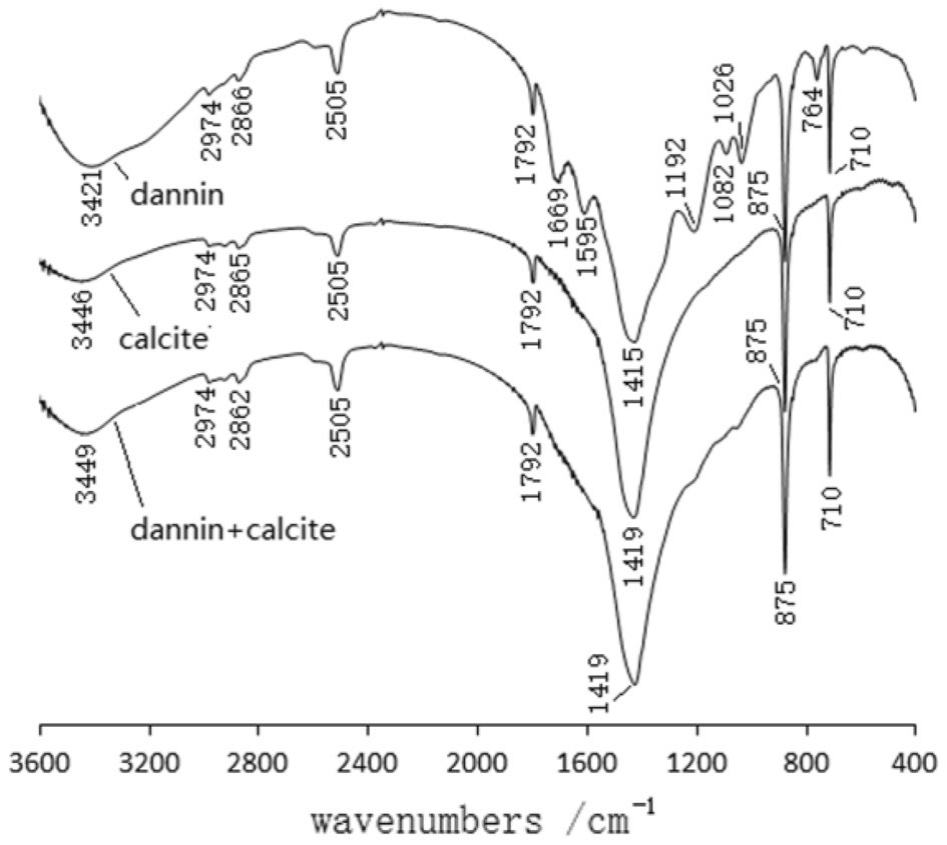
Infrared spectra of calcite, calcite and tannin with adsorbed tannins.

Figure 14 shows the IR spectra of calcite, calcite and tannin adsorbed to tannins. The IR spectra of calcite and tannin at 2505/cm, 875/cm, 710/cm, show a strong peak which is the vibrational peak of –COO^17^. The IR spectra of calcite adsorbed on tannins showed a new weak absorption peak at 1200/cm and 1036/cm compared to the IR spectra of calcite. The absorption peak at 1200/cm is the -O-vibration peak formed by the adsorption of calcite by tannins, while 1036/cm is the C–H vibration peak formed by the adsorption of calcite by tannins.

**Figure 15:**
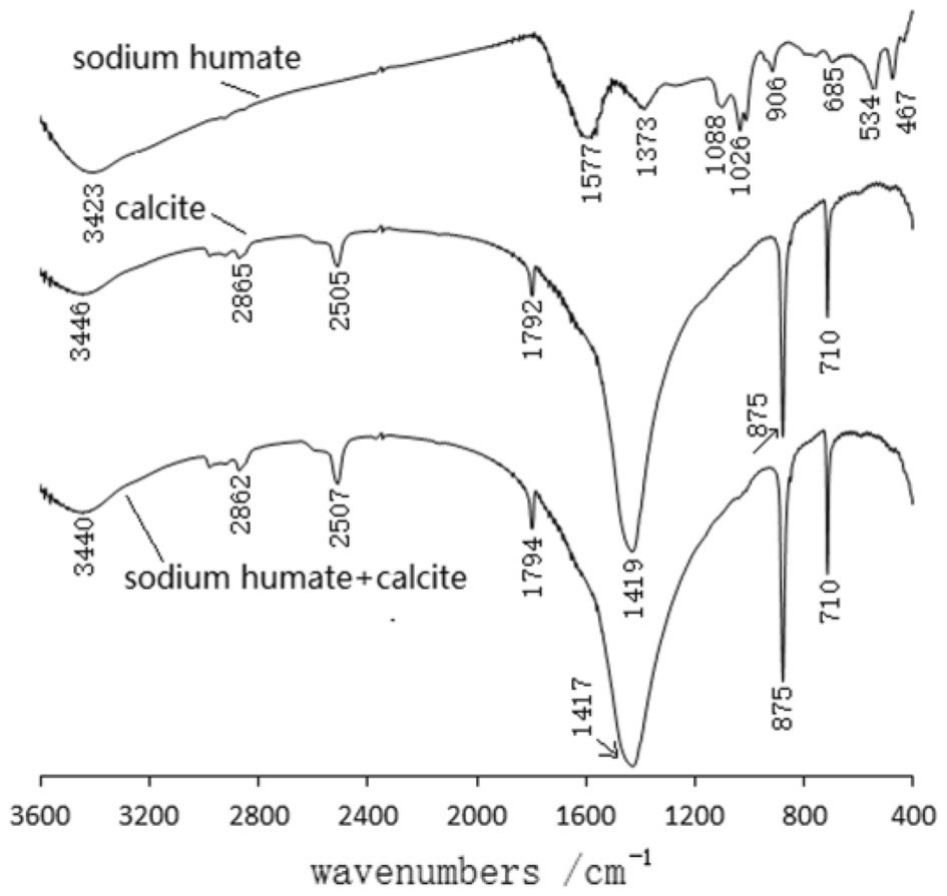
Infrared spectra of calcite, calcite and sodium humate with adsorbed sodium humate.

Figure 15 shows the IR spectra of calcite, calcite and sodium humate with adsorbed sodium humate. The IR spectra of sodium humate are at 1026/cm, 1373/cm, 1577/cm, and 3423/cm with C–O–C, C–N, C=C, and –OH stretching vibrational peaks, respectively^18,19^. Therefore, aromatic compounds such as –OH, –NH2, COOH and C–O–C were found in sodium humate. The IR spectra of calcite adsorbed by sodium humate compared to the IR spectra of calcite showed strong absorption peaks due to calcite. However, there is a new weak absorption peak at 1028/cm, which may be an asymmetric peak of the ether group (C–O–C) in sodium humate.

### Molecular simulation

As shown in Table 5, calcite adsorbed tannin and sodium humate with larger adsorption energy, and the adsorbed tannin and sodium humate were not easily detached, and the adsorption state was more stable; on the contrary, fluorite adsorbed tannin and sodium humate with smaller adsorption energy, and the adsorbed tannin and sodium humate were easily detached, and the adsorption state was not stable. Calcite is more easily adsorbed on the calcite surface under the same conditions as fluorite, both tannin and sodium humate, making calcite more hydrophilic. It is easier to selectively separate calcite and fluorite in the flotation process.

**Table 5:** Adsorption energy of calcite (1 − 1 2) surface and fluorite (1 1 1) surface after adsorption of tannin and sodium humate molecules, respectively.

System	Adsorption energy/kcal/mol
Calcite-Tannin	− 983.918527
Calcite-Sodium Humate	− 956.661501
Fluorite-Tannin	− 64.839806
Fluorite-Sodium Humate	− 13.648928

It can be seen from Table 6 that the absolute values of adsorption energy of calcite adsorbing tannin and sodium humate at the same time are higher than those of calcite adsorbing tannin alone and calcite adsorbing sodium humate alone, and the state of calcite adsorbing tannin and sodium humate existing at the same time is relatively more stable, so the effect of tannin and sodium humate synergistically inhibiting calcite is better than the effect of tannin and sodium humate inhibiting calcite alone.

**Table 6:** Adsorption energy of tannin and sodium humate molecules adsorbed on the surface of calcite (1 − 1 2) and the adsorption energy of tannin and sodium humate adsorbed on the surface of calcite (1 − 1 2) simultaneously.

System	Adsorption energy/kcal/mol
Calcite-Tannin	− 983.918527
Calcite-Sodium Humate	− 956.661501
Calcite-tannin-sodium humate	− 998.063107

## Discussion

**Figure 16:**
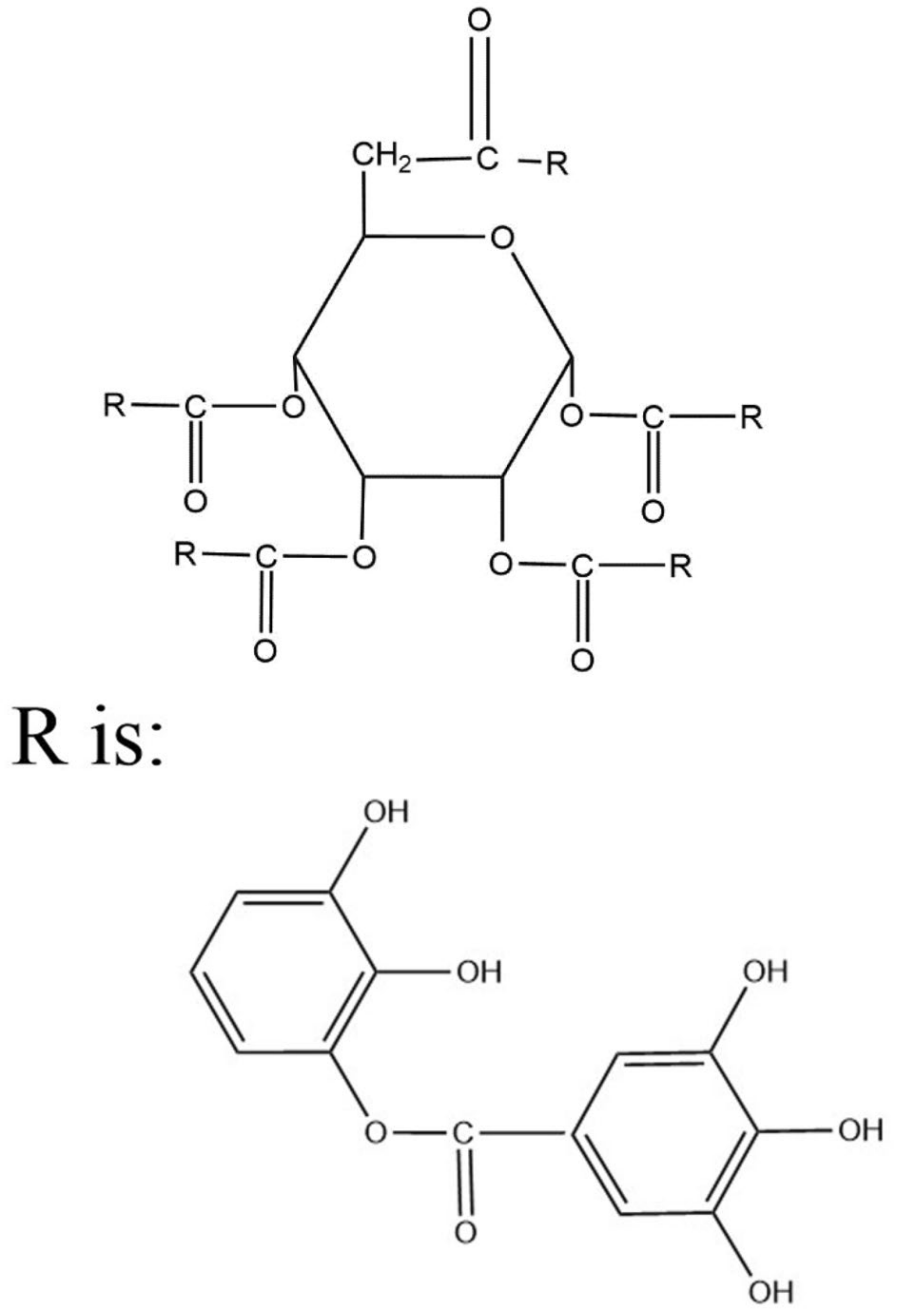
Molecular structure of tannins.

Sodium humate is mainly composed of C, O and a small amount of H, N and S. It is an amorphous macromolecular compound. Sodium humate has a large number of functional groups such as carboxyl, phenolic hydroxyl, alcohol hydroxyl, hydroxyquinone, amino, and sulfonic acid groups^20,21^. Ellagic acids are molecules with large amorphous substances, and tannins have a large number of polar groups in their molecular structure, mainly –O and –COOH^22^. Among them, the molecular structure of tannins is shown in Fig. 16.

From previous studies, it was shown that calcite has a greater adsorption capacity for tannin and sodium humate than fluorite at pH = 8. This is the reason why sodium humate and tannin can be used as depressants for fluorite flotation of calcite. Similarly, the above-mentioned study showed that sodium humate and tannins were chemisorbed on the calcite surface.

The PZCs of calcite and fluorite were 9.7 and 11, respectively. When pH = 8, both minerals are positively charged because the anions in both minerals are preferentially dissolved. Mineral surfaces have exposed Ca protons with which polar groups such as carboxyl and hydroxyl groups in the structure of sodium humates and tannins bind, complex or chelate, thus adsorbing to the mineral surface. Other polar groups not adsorbed with the mineral interact with water molecules, which makes calcite more hydrophilic and prevents the mineral surface from interacting with oleic acid.

**Figure 17:**
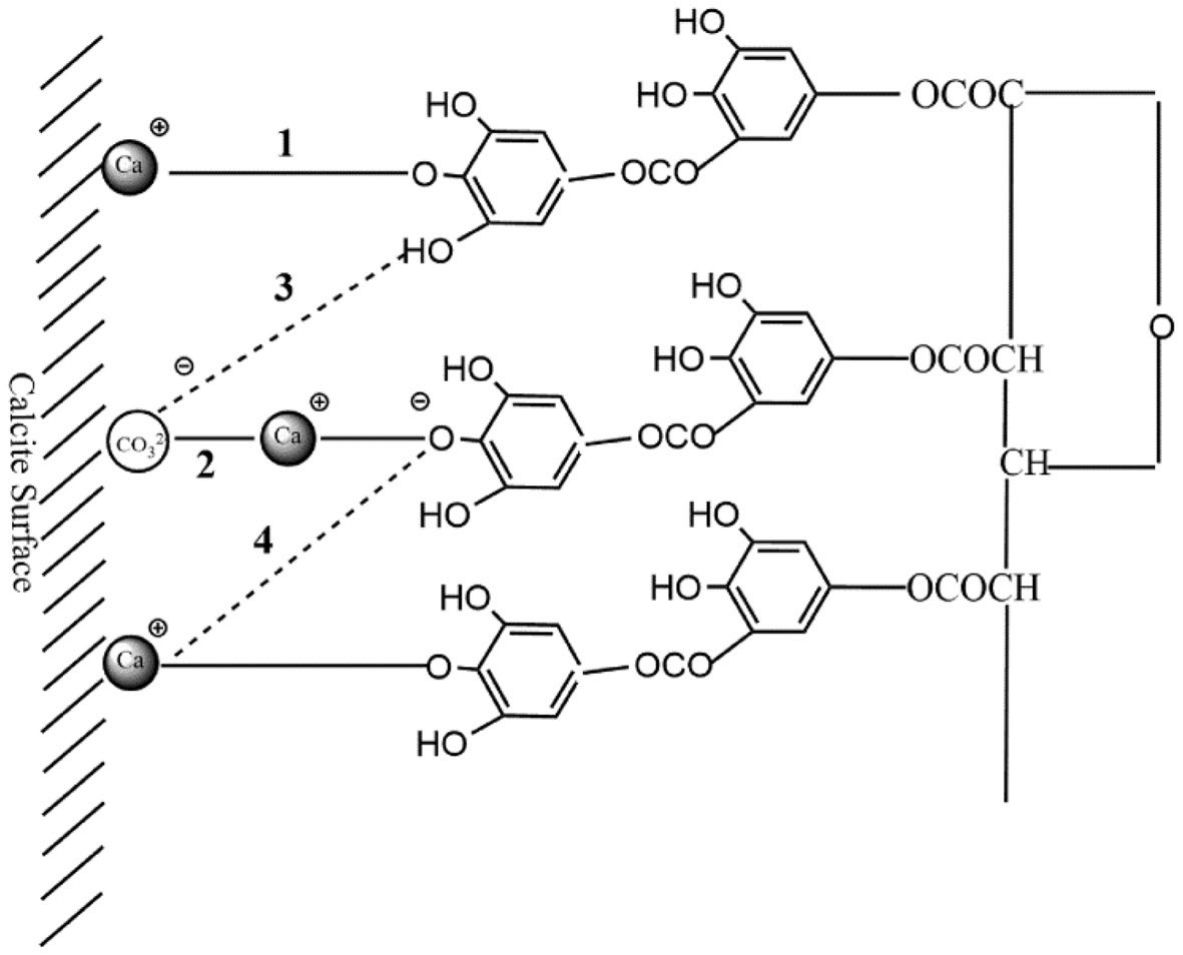
Adsorption of tannins on calcite surface. 1, Direct Ca–O bond; 2, Ca^2+^ active bond; 3, hydrogen bond; 4, electrostatic attraction.

**Figure 18:**
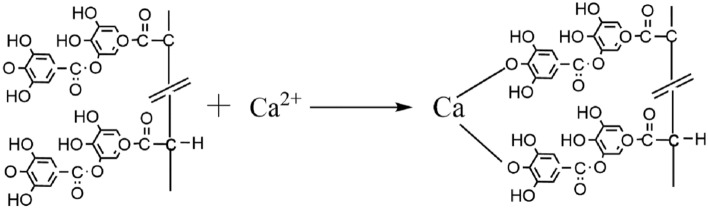
Chemical reaction of tannic acid with calcium ions.

**Figure 19:**
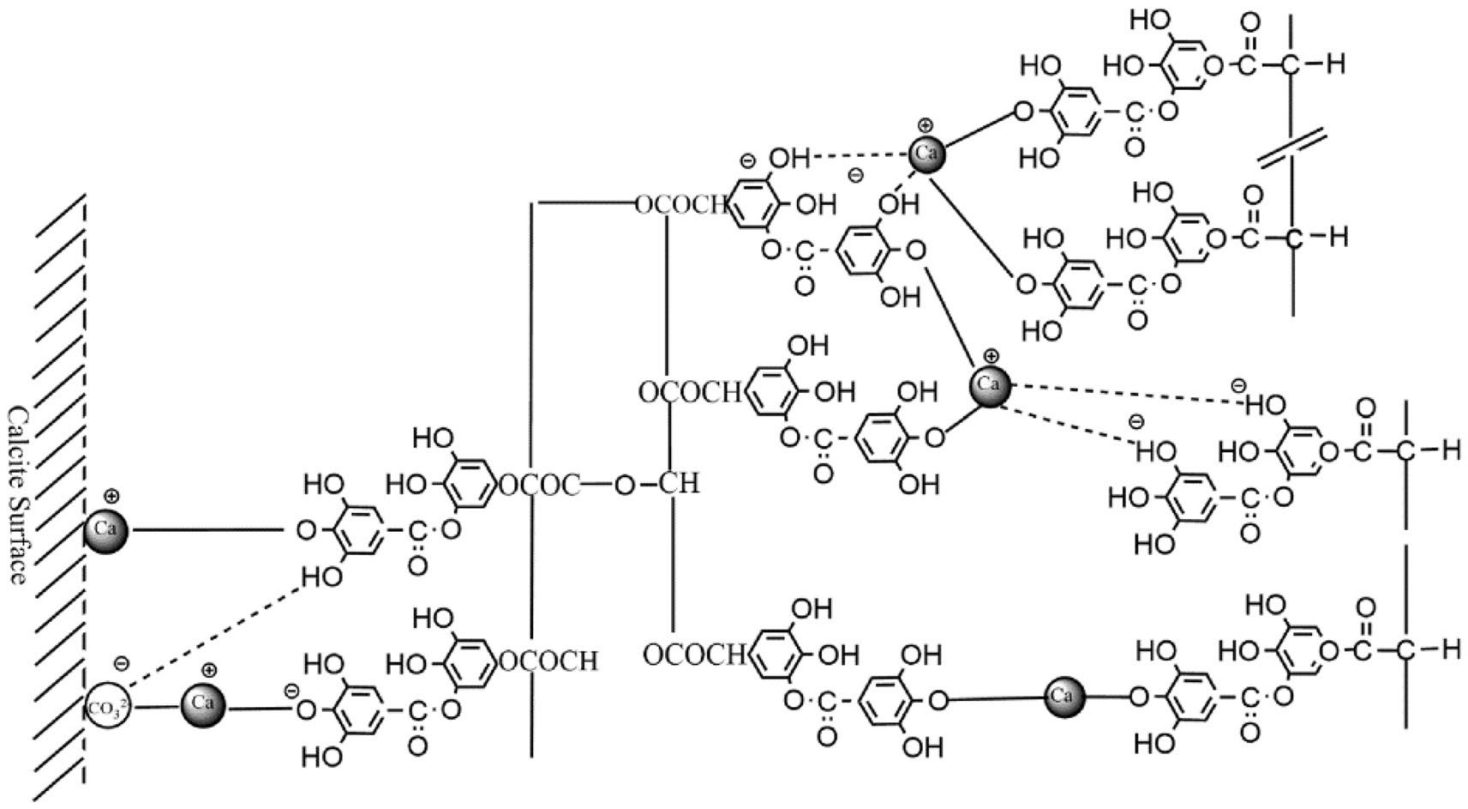
Model of multilayer adsorption of tannins on calcite surface.

The model of tannin adsorption on calcite is shown in Fig. 17^22^. Since calcite dissolves faster than fluorite and has more Ca protons on its surface than fluorite surface, the adsorption capacity of calcite surface for sodium humate and tannin is greater than that of fluorite. Also, the calcium ions dissolved from calcite are much larger than fluorite, and these ions are also chelated with sodium humate and tannic acid to form organic salts. The chemical reaction between tannins and calcium ions is shown in Fig. 18^23^. These organic salts adsorb on the organic compounds on the surface of calcite, forming multiple layers of adsorption on the surface of calcite. The presence of a large number of polar functional groups in these adsorption products further makes the surface of calcite minerals hydrophilic and hinders the adsorption of the trapping agent on calcite, thus inhibiting the flotation of calcite. The multilayer adsorption model of calcite surface is shown in Fig. 19.

## Conclusion

1. Under weak alkaline conditions, tannin and sodium humate compounds have the best depressant effect on calcite. The optimal flotation conditions for the refractory fluorite ore in Guizhou were determined through experiments. The fineness of grinding is − 0.074 mm, accounting for 75%, the amount of sodium carbonate is 1000 g/t, the amount of water glass is 300 g/t, the amount of Df01 is 100 g/t, and the amount of oleic acid is 300 g/t. Through the flotation process of primary roughing, primary sweeping and four-stage selection of fluorite ore, a qualified fluorite concentrate with CaF_2_ grade of 95.52% and recovery of 91.20% was obtained, meeting the demand for chemical tertiary fluorite concentrate.
2. Sodium humate has a strong inhibiting ability to calcite, while it has a weak inhibiting effect on fluorite flotation. Sodium humate has a good selective depressant effect on calcite. Tannin has a strong depressant effect on both minerals under alkaline conditions at pH values greater than 9. In the pH range of 6–8, it is stronger than fluorite for calcite.
3. Sodium humate and tannins are chemisorbed on the surface of calcite. The adsorption capacity of tannin and sodium humate on calcite was greater than that of fluorite at pH 8. Adsorption of the reagents resulted in a negative surface dynamic potential for calcite and fluorite, and the negative value increased with increasing pH. After the adsorption of tannins on calcite, new chemical bonds with –O- and C–H appear on the calcite surface. After calcite adsorption, a new absorption peak also appeared on the calcite surface.
4. Polar groups in the molecules of sodium humate and tannins, such as carboxyl and hydroxyl groups, bind, complex or chelate with calcium ions on the surface of calcite. At the same time, sodium humate and tannins react with calcium ions in solution to produce organic calcium salts. These calcium salts interact with organic compounds adsorbed on the calcite surface, forming multiple layers of adsorption on the calcite surface, thus making the calcite more hydrophilic and hindering the adsorption of the trapping agent.
5. Based on density flooding theory, Materials Studio (MS) software was used to calculate the adsorption energy associated between these molecules of calcite, fluorite, tannin, and sodium humate, and the results were obtained as follows: (a) Tannin molecules and sodium humate molecules are more easily adsorbed on the surface of calcite compared to fluorite. (b) Compared to calcite adsorption of tannin molecules or sodium humate molecules alone, simultaneous adsorption of both states would be more stable, and tannin and sodium humate synergistically inhibited calcite better than alone.

## Data Availability

All data generated or analyzed during this study are included in this published article.
